# “The ubiquitin ligase SIAH2 is a female-specific regulator of circadian rhythms and metabolism”

**DOI:** 10.1371/journal.pgen.1010305

**Published:** 2022-07-05

**Authors:** Tsedey Mekbib, Ting-Chung Suen, Aisha Rollins-Hairston, Kiandra Smith, Ariel Armstrong, Cloe Gray, Sharon Owino, Kenkichi Baba, Julie E. Baggs, J. Christopher Ehlen, Gianluca Tosini, Jason P. DeBruyne

**Affiliations:** 1 Neuroscience Institute, Department of Pharmacology and Toxicology, Morehouse School of Medicine, Atlanta, Georgia, United States of America; 2 Neuroscience Institute, Department of Neurobiology, Morehouse School of Medicine, Atlanta, Georgia, United States of America; University of Florida College of Medicine, UNITED STATES

## Abstract

Circadian clocks enable organisms to predict and align their behaviors and physiologies to constant daily day-night environmental cycle. Because the ubiquitin ligase *Siah2* has been identified as a potential regulator of circadian clock function in cultured cells, we have used SIAH2-deficient mice to examine its function *in vivo*. Our experiments demonstrate a striking and unexpected sexually dimorphic effect of SIAH2-deficiency on the regulation of rhythmically expressed genes in the liver. The absence of SIAH2 in females, but not in males, altered the expression of core circadian clock genes and drastically remodeled the rhythmic transcriptome in the liver by increasing the number of day-time expressed genes, and flipping the rhythmic expression from nighttime expressed genes to the daytime. These effects are not readily explained by effects on known sexually dimorphic pathways in females. Moreover, loss of SIAH2 in females, not males, preferentially altered the expression of transcription factors and genes involved in regulating lipid and lipoprotein metabolism. Consequently, SIAH2-deficient females, but not males, displayed disrupted daily lipid and lipoprotein patterns, increased adiposity and impaired metabolic homeostasis. Overall, these data suggest that SIAH2 may be a key component of a female-specific circadian transcriptional output circuit that directs the circadian timing of gene expression to regulate physiological rhythms, at least in the liver. In turn, our findings imply that sex-specific transcriptional mechanisms may closely interact with the circadian clock to tailor overt rhythms for sex-specific needs.

## Author summary

Circadian clocks in our bodies align all processes to each other as they vary in function across the solar day. One of the clearest examples is that the clock aligns internal metabolic processes with the daily rhythms in sleep/wake cycles and feeding/fasting cycles. Disruptions in the alignment between these internal metabolic rhythms and behavioral feeding/fasting rhythms, such as eating at the ‘wrong time of day’, leads to obesity and other metabolic disorders. Here, we report data that reveal the *Siah2* gene plays a key role in orchestrating the proper circadian timing of metabolic gene expression and overall metabolism, but, surprisingly, only in females. This role of Siah2 appears distinct from processes that are known to be different between males and females, and suggest for the first time that how the circadian clock regulates metabolism in females is mechanistically distinct from males. This is particularly important as nearly all mechanistic studies on metabolism are focused on males, thus opening the door for the need to further develop tailored sex-specific therapeutic targets and approaches, as those that work for males may not work for females.

## Introduction

Circadian rhythms in physiology and behavior are driven by a transcriptional feed-back loop timing mechanism that drives ~24 hour rhythms in expression of 1,000’s of target genes [1–3] thoughout the body. How circadian clocks drive these rhythms is thought to be due to largely similar transcriptional pathways and mechanisms in males and females, although some rhythms are modulated by sex and growth hormones. Disruption of these rhythms, or more commonly, misalignent of these rhythms with the environmental day-night cycle or within the organism causes a wide-range of health consequences, including metabolic dysfunction and obesity [4–6].

SIAH2 is a ring-type E3 ubiquitin ligase whose role in regulating the hypoxia pathway [7] and tumorogenesis is well known [8,9]. We have recently reported evidence suggesting that SIAH2 is also a regulator of circadian clock function [10]. We identified SIAH2 in a screen for ubiquitin ligases that mediate degradation of REVERBα/β, heme-sensitive transcriptional repressors that regulate circadian rhythms and lipid metabolism [11–17]. Suppressing *Siah2* expression in a cellular clock model U2OS cells both altered REVERBα stability and lengthened periodicity, thus directly implicating SIAH2 in the regulation of circadian rhythms [10]. To explore this possibility further, we have now examined the effect of SIAH2 deletion on clock function in a mouse model [18]. Here we present data that reveal SIAH2 is a component of unexpectedly female-specific transcriptional mechanisms that are essential for the proper rhythmic control of gene expression in the liver. We also found that disrupting this mechanism substantially impairs the circadian regulation of lipid and cholesterol metabolism selectively in females and weakens their resistance to diet-induced obesity, suggesting sex-specific circadian mechanisms may contribute broadly to differences in male and female physiology.

## Results

**Fig 1:**
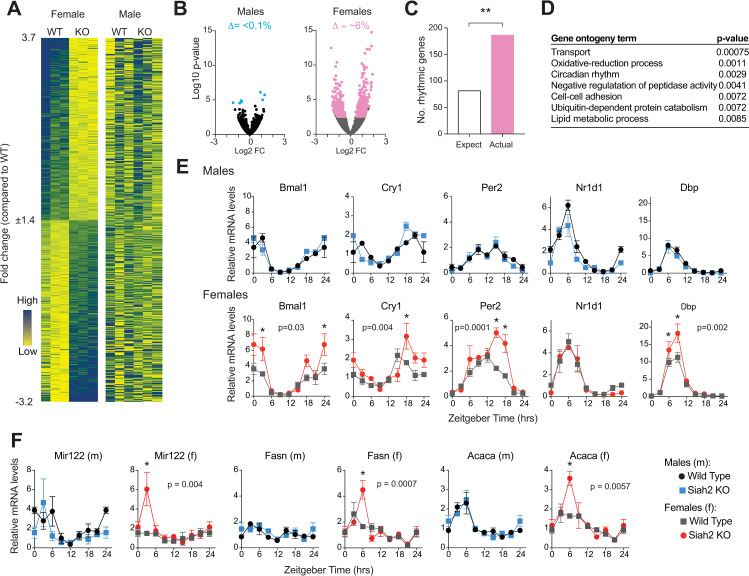
SIAH2 loss preferentially alters daily gene expression in females. **A**. Heatmaps depicting the expression of the genes affected by SIAH2 loss in female livers harvested at ZT10 in all four groups. FDR corrected p-value <0.05 was used as a cutoff. Each gene is aligned across genotype (WT = wild type, KO = *Siah2*^*-/-*^) and sex. **B**. Volcano plots comparing hepatic gene expression changes within sex (n = 3/group). Colored transcripts are significantly changed by SIAH2 loss in male (blue) or females (pink) as determined by DESeq2 (adj p <0.05). **C**. Enrichment of rhythmically expressed genes among those altered by SIAH2 loss in females, using 16% as the expectation for rhythmic genes (http://circadb.hogeneschlab.org/mouse, Ref 19; * = p<0.0001 Fisher’s exact test). **D**. DAVID gene ontogeny of the rhythmic genes altered by SIAH2 loss in females. Also see S1 Dataset. **E-F** Quantitative RT-PCR profiles of clock and REVERBα target gene mRNAs in liver (mean +/- sem, n = 3 livers per genotype, sex and time.). The Zeitgeber time (ZT) 0 point is double plotted at ZT24 for clarity. ZT0-12 = lights on, ZT 12–24 = lights off. P-values shown reflect significant time x genotype interactions (two-way ANOVA). * = p<0.05 between genotypes at individual timepoints (Sidak’s multiple comparison test).

REVERBα protein levels reach peak abundance levels during the daytime in most tissues, and in the liver, they reach peak levels around Zeitgeber time (ZT) 9–10, which is ~2–3 hours before lights go out (lights off = ZT12; 14). Since REVERBα functions as a transcriptional repressor, we first asked if there were detectable changes in gene expression in livers harvested from SIAH2 KO mice just after REVERBα peak levels using a QuantSeq 3’mRNA counting approach. In this experiment, we obtained mRNA abundance data from individual livers, obtained from three mice of each sex and genotype (*Siah2*^*-/-*^ and wild type, 12 mice in total). Intriguingly, we did not detect any significant changes in expression when data from both sexes were combined. However, when we compared the effect of SIAH2 loss within each sex, we found that it altered the levels of ~6% (513 of 8709) of transcripts compared in livers of female mice, but less than 0.1% (7 of 8405) of transcripts compared in males (Fig 1A and 1B; see also S1 Dataset). This unexpected predominantly female-specific effect further piqued our interest as the transcripts altered by SIAH2 loss in females appear to be enriched by >2-fold for genes that are under circadian control in the male liver (Fig 1C) [19], and included some genes associated with circadian rhythms and processes that can be regulated by the circadian clock (i.e. redox, lipid metabolism) (Fig 1D). Thus, despite that these data were from a minimum number of mice harvested at a single time point, they suggested a hypothesis that SIAH2 loss may have a larger impact on circadian regulation in females.

To explore this possibility, we examined the expression of five core rhythmic genes (*Bmal1*, *Cry1*, *Per2*, *Nr1d1/RevErbα* and *Dbp*) [1–3] and several REV-ERBα transcriptional targets [11,12,17,20] in the livers of both male and female SIAH2-deficient and wild type mice. Expression was examined at 3-hour intervals throughout a 24-hour period. We found a striking difference in the effect of *Siah2* gene deletion between female and male mice (Fig 1E). In males, loss of SIAH2 has no detectable effect on the expression of any of these genes. In female mice, however, SIAH2 loss increased the peak expression of several core clock genes (Fig 1E) and delayed the expression of the repressors *Per2* and *Cry1* (Fig 1E). Expression of several REV-ERBα targets was also altered by SIAH2-deficiency selectively in females (Fig 1F). The effects were not limited to a specific time of day–SIAH2 loss increased and/or delayed peak expression of these genes regardless of the time of day each is normally maximally expressed, suggesting that the circadian rhythm amplitude may be broadly enhanced in female livers without SIAH2. These results are somewhat different to our previous results in U2OS cells (which are female [21]) where SIAH2 knockdown blunted expression of REVERBα targets coincident with altered REVERBα protein turnover [10]. However, in the liver, we were surprised to find that SIAH2 loss had little effect on rhythms of REVERBα protein abundance rhythms of either sex (S1 Fig), possibly owing to tissue-specific compensation by other ubiquitin ligases [22–24]. Thus, it is not clear if these effects of SIAH2 loss are through alterations in REVERBα function, though female-specific regulation of REVERBα is not expected given its role in circadian regulation. Nonetheless, the effect on gene expression in female livers suggests that SIAH2 broadly regulates the amplitude and timing dynamics of circadian gene expression *in vivo*, but only in females.

We next examined the effect of SIAH2 loss on the entire hepatic transcriptome via RNAseq on livers harvested around the clock. For this experiment, we chose to employ a design similar to one used by Chaix et al [25] that sought to strike a balance between costs, number of animals, and time resolution and would enable us to compare the consequences of SIAH2 loss in males and females. We therefore assayed the transcriptomes using RNAs pooled from three livers harvested from each sex and genotype at 3-hour intervals across a single day (representing 96 mice) from above (Fig 1E and 1F) using RNAseq. While we anticipated that pooling may obscure some of the single-timepoint differences observed using qRT-PCR above, this design would allow us to identify broader changes in the patterns of daily expression, reflected by substantial differences across multiple timepoints among the four groups, while simultaneously buffering against variability between individuals.

**Fig 2:**
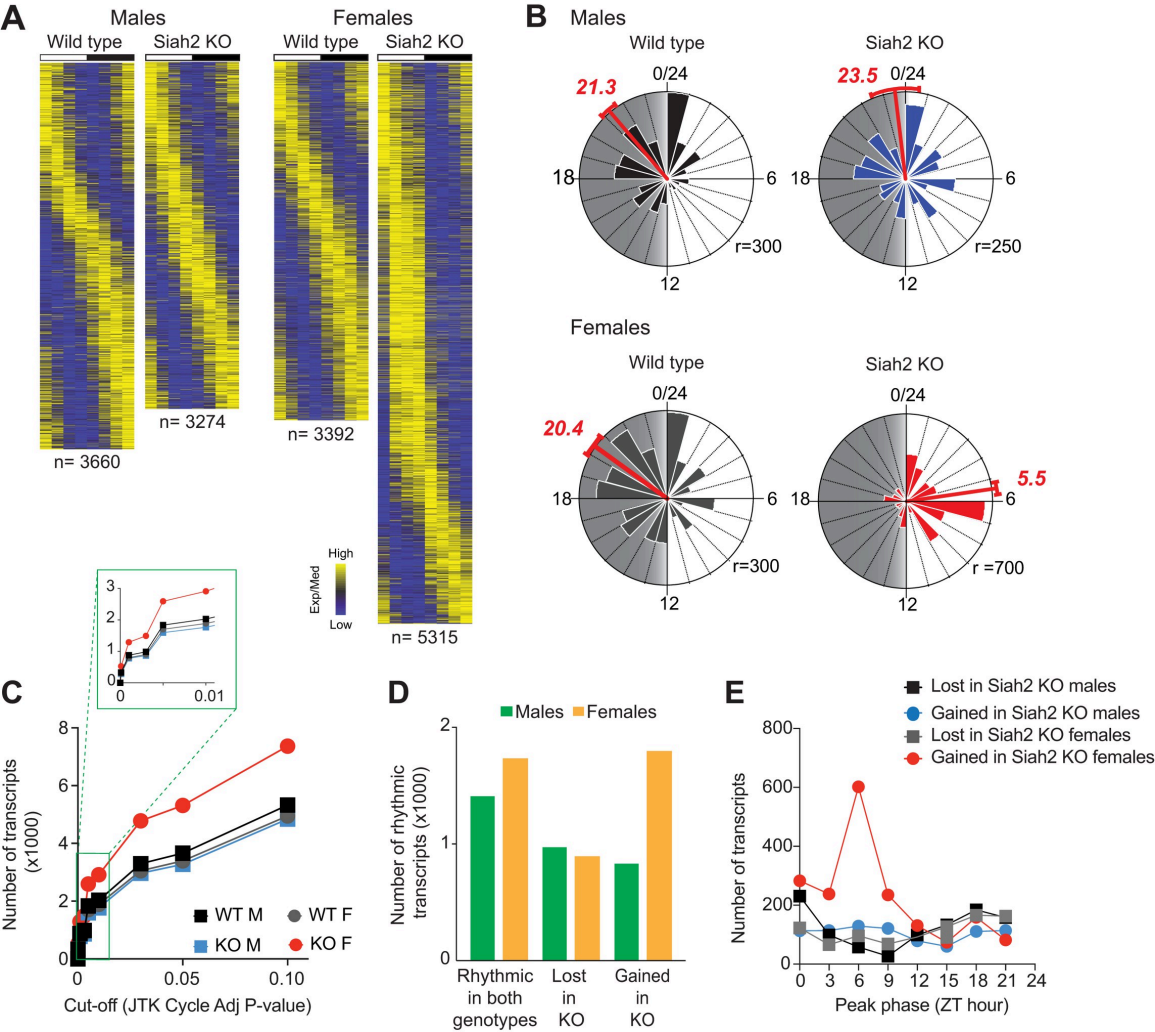
SIAH2 loss selectively remodels the female circadian transcriptome towards the daytime. (**A**) Heatmaps of expression profiles of the rhythmically expressed genes (JTK cycle adj p<0.05), sorted by peak expression timing/phase for each group independently. White/black bars indicate the light-dark cycle. (**B**) Raleigh plots (circular histograms) of expression peak timing for all rhythmically expressed genes in each group. The numbers in red depict the average peak-time (in ZT hours) indicated by vector analysis (red line, +/- 95% CI). Gray shading = nighttime, r = radius in number of genes. **C.** Numbers of ‘rhythmically’ expressed genes in each group at various statistical cut-offs. (**D**) Comparisons of SIAH2-induced changes to rhythmicity of genes in each sex. (**E**) Frequency distribution of expression peak timing, across the day, of genes that gained or lost rhythmicity in SIAH2-deficient (Siah2 KO) livers in both sexes.

Remarkably, results from this experiment revealed that SIAH2 loss in females drastically reorganized the timing of a rhythmic gene expression in livers. In both wild type males and females, most rhythmically expressed genes peaked during the night, with a population mean vector of ZT ~20–21 (Fig 2B). In *Siah2*^*-/-*^ males, the global rhythmicity pattern appears mildly shifted (~2 hours) towards dawn. In females however, SIAH2 loss shifted the the global expression profile by ~9 hours (Fig 2B), with the vast majority of rhythmic genes peaking during the daytime instead of the night. This near inversion of rhythms in female *Siah2*^*-/-*^ livers strongly suggests SIAH2 may play a central role in organizing circadian rhythms in gene expression, predominantly in females.

This female-specific reorganization of the rhythmic transcriptome is the consequence of two principal changes. First, SIAH2 loss in females resulted in a dramatic net increase (ca. 50–65%) in the number of rhythmically expressed transcripts regardless of how we defined ‘rhythmicity’ (Fig 2C; see also S2 Fig and Materials and Methods). This increase was due to a larger population of genes that gained rhythmicity in SIAH2-deficient females over genes that lost rhythmicity (Fig 2D). In contrast, SIAH2-deficiency in males caused a small net decrease (ca. 7%) in the overall number of rhythmic genes. Strikingly, most of the genes that gained rhythmicity in *Siah2*^*-/-*^ females did so with peak expression strongly clustered around mid-day (ZT6, Fig 2E), accounting for part of the overall shift in timing from night time to daytime. In contrast, changes in gene expression in males lacked phase clustering (Fig 2E). The large bias in the timing of expression of genes in females suggests that SIAH2 may regulate a female-specific transcriptional mechanism that regulates gene expression specifically around mid-day.

**Fig 3:**
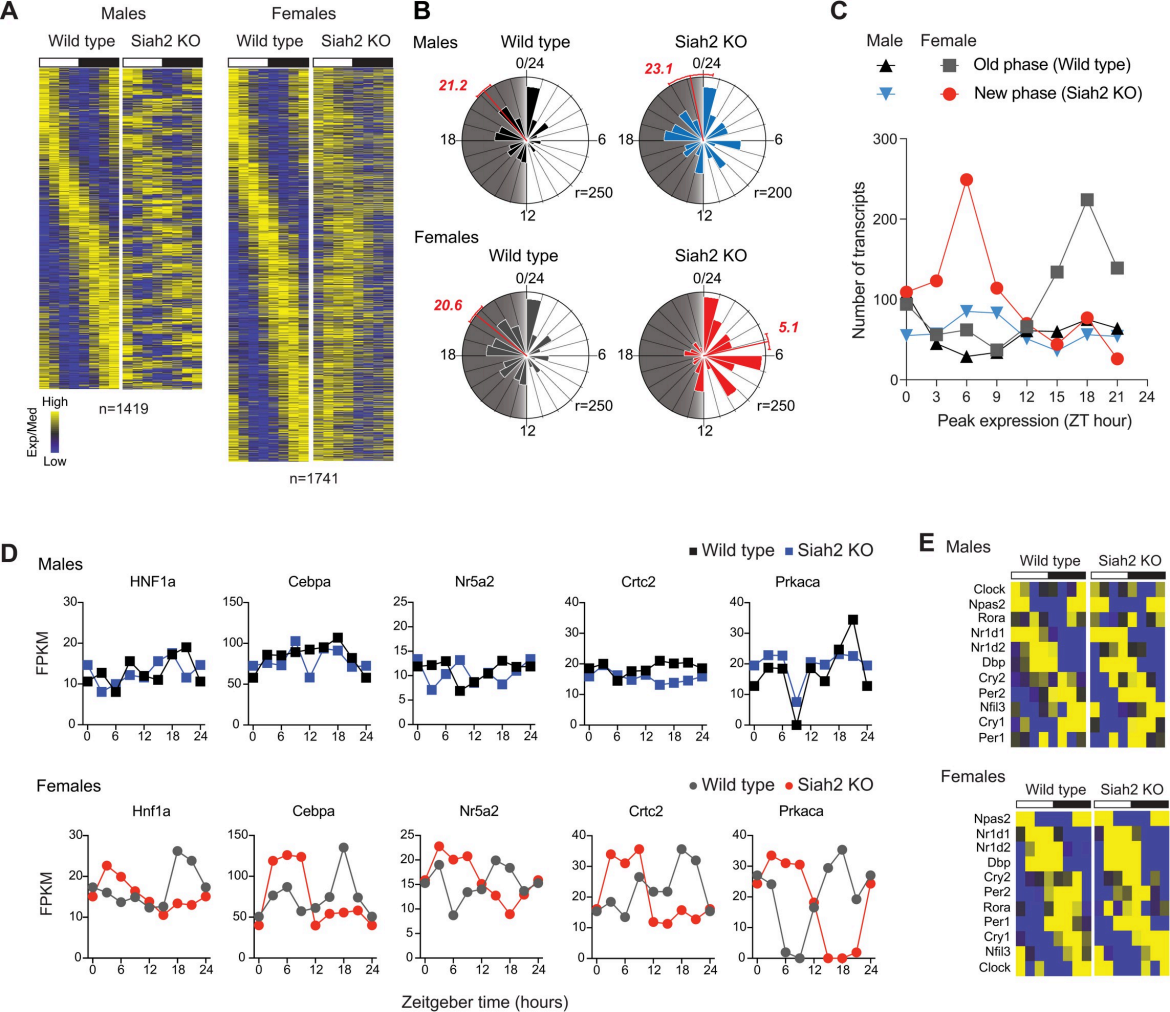
SIAH2 loss in females shifts rhythmic gene expression profiles from night to day. (**A**) Heatmaps of genes that were rhythmically expressed in both genotypes; genes are aligned across genotypes (but not across sex). (**B**) Raleigh plots of the peak expression phase for the genes in A. Plotted as decribed for Fig 2B. (**C**) Frequency distributions of peak phase across time of day for genes rhythmically expressed in both genotypes but shifted by more than 6 hours between genotypes. See also **S1 Fig** and **S2 and S3 Datasets**. (**D**) Heatmaps depicting similar rhythmic expression of core circadian clock genes across all four groups. (**E**) Example gene expression profiles of genes across all four groups.

Second, SIAH2 loss nearly eliminated the number of transcripts with peak expression during the night in wild type females by shifting their expression profile to peak during the day (Fig 3). Examination of the patterns of genes that were rhythmically expressed in both genotypes revealed that SIAH2 loss changed the time of peak expression levels of individual genes in both sexes (Fig 3A). In males, SIAH2-deficiency shifted expression of genes that are expressed at all times of day, and did not alter the liver’s overall gene expression profile (Fig 3A and 3B). In surprising contrast, SIAH2 loss in females predominantly shifted genes expressed during the night in wild type livers to peak during the day in *Siah2*^*-/-*^ livers (Fig 3A and 3B).

Since these genes are rhythmically expressed in both genotypes, this change is the result of a phase shift in the timing of expression, in many cases by 12 hours. To quantify this effect, we calculated the number of genes whose expression phase was shifted by at least 6 hours as a function of their peak phase in each genotype, for both males and females (Fig 3C). This analysis demonstrated that SIAH2 loss had a remarkable time-of-day specificity in its effect. Genes that normally peak around midnight (ZT18) in wild type female mice were preferentially shifted by 6 or more hours in SIAH2-deficient females to now peak around midday (ZT6; Fig 3C). The result is a dramatic increase in the number of genes with peak expression during the day and reduction in the number of genes whose expression peaked during the night. Surveying a number of individual gene profiles (Fig 3D) revealed that SIAH2 loss in female livers both increased expression of affected genes during the day but suppressed their expression during the night. This suggests that, in females, SIAH2 has precisely timed opposing functions–limiting/repressing expression of target genes around mid-day while also promoting their expression at night.

The timing in expression of core clock genes was not drastically shifted in our transcriptome data from *Siah2*^*-/-*^ KO females (Fig 3E) and largely similar with our initial data (Fig 1E). However, the transcriptome reorganization did include 100’s of transcription factors (Fig 3D, see also S2 Dataset), suggesting SIAH2 regulates a downstream transcriptional network. Similarly, although changes in locomotor and feeding rhythms can drastically alter rhythmic gene expression in the liver by driving systemic circadian cues, these behavioral rhythms were not detectably altered by SIAH2 loss (S4 Fig). Thus, the shift in rhythmic gene expression in *Siah2*^*-/-*^ females is not due to an overall change in the core circadian clockwork, either systemically or within the liver. Surprisingly, the transcriptome reorganization in *Siah2*^*-/-*^ females appears to be specific to the ‘rhythmicity’ of gene expression, as we found only 13 genes whose expression was globally altered by SIAH2 loss if time-of-day was not used as a variable (S3A Fig). These findings imply that the role of SIAH2 in directing rhythms in gene expression is likely through regulation of a female-specific transcriptional network intrinsic to the liver and regulated by the circadian clock while also possibly providing feedback to regulate amplitude of core clock gene expression.

Since the *Siah2* gene is not X-linked and its expression is comparable between male and female livers (S3B Fig), we looked for evidence to suggest that SIAH2 regulates known sexually dimorphic transcriptional pathways in the liver. We found considerable differences in rhythmicity between wild type males and females, but the effect of SIAH2 loss in females was clearly distinct from these normally dimorphic differences (S3C Fig). In addition, we found that SIAH2 KO females have normal levels of sex-hormones (S3D Fig), homozygous *Siah2*^*-/-*^ pairs breed as well as wild type pairs (S3E Fig), and genes that are regulated by estrogen signaling were not enriched among those altered by SIAH2 loss (S3F Fig) [26]. Similarly, SIAH2 loss did not alter expression of key mediators of growth hormone signaling in the liver, another major source of sexually dimorphic gene expression in the liver [27–29] (S3G Fig; see also S2–S5 Datasets). Taken together, we did not find any evidence to suggest that SIAH2 loss impaired or altered one of these known sexually dimorphic pathways suggesting that SIAH2 likely acts within a novel female-specific transcriptional pathway to regulate rhythmic gene expression.

**Fig 4:**
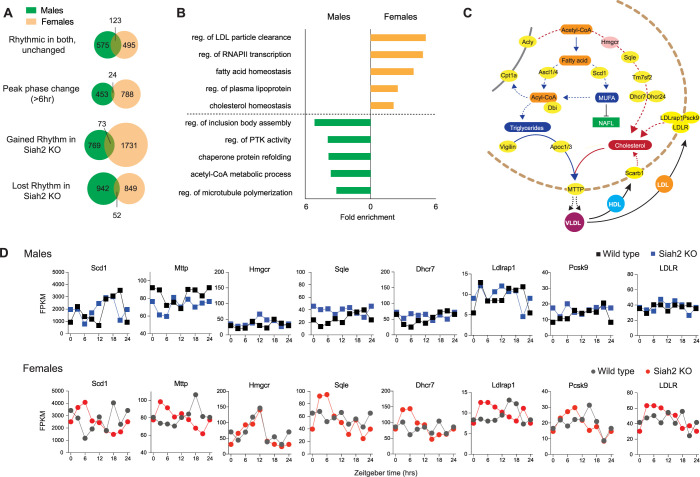
SIAH2 regulates rhythmic expression of lipid/lipoprotein metabolism genes in female livers. (**A**) Venn diagrams comparing the effects of SIAH2 loss between males and females on rhythmic gene expression. (**B**) Top gene ontogeny terms corresponding to the genes whose expression was affected by SIAH2 loss in both sexes. (**C**) Simplified lipid and cholesterol metabolic pathways in the liver, highlighting genes (yellow) uniquely affected by SIAH2 loss in females. (**D**) Examples of female-specific effects of SIAH2 loss on rhythmic gene expression. See also **S2 and S3 Datasets**.

In keeping with the strongly dimorphic role of SIAH2, we found very little overlap in the genes who’s rhythmic expression was affected by SIAH2 loss between males and females (Fig 4A). Analysis of biological function using gene enrichment tools predicted that SIAH2 has very different roles between the sexes (Fig 4B). In females, genes involved in regulating cholesterol/lipoproteins, fatty acid/lipids and gene expression (as noted above), were highly enriched among those altered by SIAH2 loss. As illustrated in Fig 4C, SIAH2 loss impacts the rhythmic expression of genes that function at multiple levels of lipid/lipoprotein regulation, including their synthesis, release and re-uptake into the liver (Fig 4C and 4D), indicating that SIAH2 has a broad impact on circadian regulation of these pathways in females. Interestingly, although expression of the *Hmgcr* gene (encoding HMG-CoA reductase, the rate limiting enzyme in cholesterol production and target of statin drugs) is not affected by SIAH2 loss, its rhythmic expression is sexually dimorphic (Fig 4D), suggesting potentially important sex-differences in the dynamic regulation of this clinically important pathway. In striking contrast, gene enrichment analysis of data from males predicted a different, almost non-overlapping set of biological functions for SIAH2 in males (Fig 4B). Combined, these analyses led us to predict that SIAH2 loss selectively impairs daily and overall lipid/lipoprotein metabolism in females. If so, then it would follow that the consequences of the reorganized rhythmic transcriptome in *Siah2*^*-/-*^ females likely creates a chronic misalignment between metabolic and behavioral locomotor and feeding rhythms, a condition that often causes obesity and other metabolic disorders [6,30–33].

**Fig 5:**
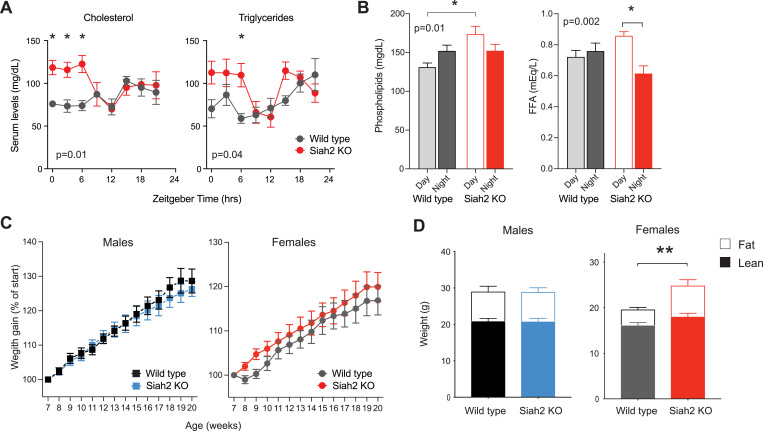
SIAH2 loss alters lipoprotein and lipid metabolism in females. (**A-B**) Serum lipid levels across the day in female mice fed normal chow *ad libitum*. In A, each point is the mean +/- SEM (n = 3 mice, except ZT 9, n = 4). In B, times 0–9 levels were averaged for day (+/- SEM, n = 13 each), times 12–21 averaged for night (+/- SEM, n = 12 each). The p-values shown denote significant genotype x time interactions from two-way ANOVA. * = p<0.05 between genotypes (A) or times of day (B) using Sidak’s multiple comparison test. (**C**). Weight gain in mice fed control diet (CD) Mean +/- SEM, n = 8–10. (**D**) Body composition analysis. Mean +/- sem, n = 4–5 mice/sex and genotype. Loss of SIAH2 significantly increased adiposity in females on control diet for 13 weeks (** = p = 0.0247, two-tailed t-test).

We first asked if the time-dependent changes in the expression of lipid-metabolic genes in *Siah2*^*-/-*^ females led to alterations in the rhythmic profiles of serum lipoproteins and fatty acids under normal conditions. We assayed serum of mice collected around the clock and found that female, but not male, *Siah2*^*-/-*^ mice displayed robust increases in serum cholesterol, triglyceride and phospholipid levels during first half of the day (Figs 5A, 5B and S5). Thus, it appears that the changes in expression of at least some of the genes altered by SIAH2 loss in females has physiologically detectable effects. Importantly, the magnitude of the changes in lipoprotein levels (elevated by ca. 20–60% in *Siah2*^*-/-*^ females compared to wild types) are very similar to or exceed those reported for *Clock*^*Δ19/Δ19*^ (elevated by ca. 15–20%) [34] and *Bmal1* KO mice (elevated by ca. 30–40%) [35,36] in which the circadian clockwork is severely compromised. In addition, serum free fatty acid (FFA) levels were slightly increased throughout the day and decreased across the night, creating a significant diurnal rhythm in FFA levels in female *Siah2*^*-/-*^ mice (Fig 5B). Since, serum cholesterol and triglycerides levels are almost exclusively regulated by the liver, these results provide strong evidence of a direct causal link between SIAH2’s role in regulating rhythmic gene expression and physiological rhythms in female livers.

The daytime specific increases in serum lipids and lipoproteins occur when mice are predominantly sleeping, creating a potential mismatch of energy availbility (serum lipids) and utilization (low physical activity). As noted above, this broadly alters metabolic regulation, often leading to increases in adiposity and obesity. We tested this as part of the next experiment (see below) by monitoring weight gain and body composition changes throughout young adulthood. We found that while there was not a detetable difference in overall body weight at 20 weeks of age in mice maintainted on normal diets (Fig 5C), *Siah2*^*-/-*^ females doubled their body fat content compared to wild type females over the same timespan (Fig 5D). *Siah2*^*-/-*^ males, in contrast, were not different from wild type. Thus, the loss of SIAH2 alters fat metabolism in relatively young adult in female, but not male, mice kept under normal conditions.

This effect is consistent with the circadian misalignment in *Siah2*^*-/-*^ females and suggests that they may be more susceptible to developing obesity. In male mice, similar circadian misalignments are proposed to be prominent causes of obesity and metabolic disorders in mice, most of which are made more readily detectable when fed high-fat diets [6,25,30–32]. High-fat diets (HFD) are widely used to exacerbate differences in fat metabolism that underlie development of obesity and other aspects of metabolic syndrome (i.e. diabetes, hepatic steatosis). Importantly, HFD studies are almost exclusively performed using male mice because female mice are resistant to the development of HFD-induced obesity and development of other metabolic consequences due to, at least partially, complex interactions between sex hormones and chromosomes [33,37–39]. However, since our data indicate that SIAH2 loss alters gene expression rhythms and the metabolism of lipids/lipoproteins specifically in female mice, we asked if SIAH2 loss similarly makes females more prone to developing obesity and other metabolic disorders when fed HFD.

**Fig 6:**
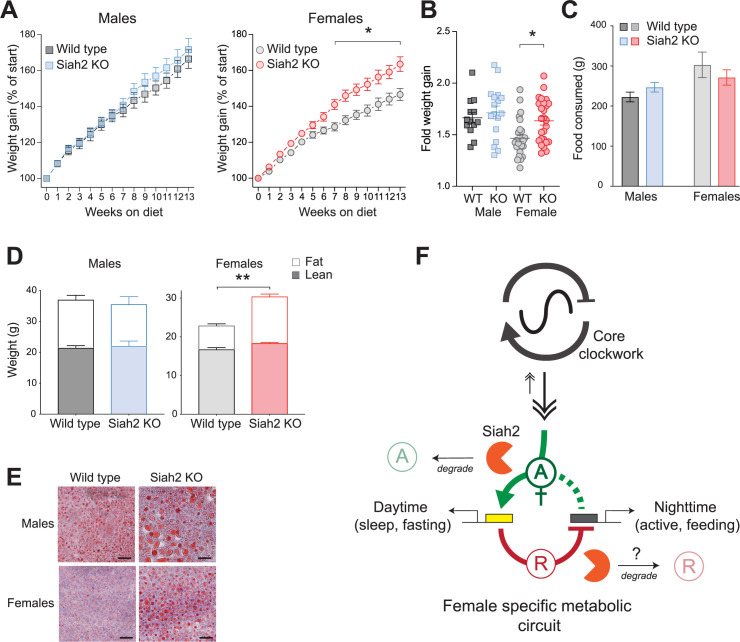
SIAH2 protects females from diet-induced metabolic disorders. (**A**) Weight gain in mice fed high fat diet (45% fat). n = 13–17 male mice, combined from two trials, or 26–27 female mice from 4 trials. Two-way ANOVA indicated a significant interaction between genotype x time on diet in females only (p<0.0001, F (13, 663) = 6.484), with significant differences in weight gain by week 7 (* = p <0.01, Sidak’s multiple comparison test). (**B**) Fold weight gained at 13 weeks for the mice in **A** (* p <0.0001, t = 4.809, df = 714). (**C**) Consumption of HFD over the experiment; no statistical differences between any groups. (**D**) Loss of SIAH2 significantly increased adiposity in females on HFD for 13 weeks (** p = 0.0044, t = 4.13, DF = 15). (**E**) Oil Red O staining of liver from mice fed HFD for >13 weeks. Red staining = fat. Scale bar = 50 microns. (**F**) Model depicting how SIAH2 may regulate rhythmic gene expression in females. Our data suggest SIAH2 may, in a female-specific manner, regulate one or more transcriptional activators (green) and/or repressors (red), perhaps connected in a circuit or loop (dashed green line) that normally links the circadian clockwork to metabolic control in females.

We found that female, but not male, *Siah2*^*-/-*^ mice diplayed robust metabolic phenotypes when switched to HFD (Fig 6). In these experiments, mice were raised on standard chow until 7–8 weeks of age (ca. adolescence) before being being switched to a HFD (45 kCal from fat). Remarkably, after 7 weeks on HFD (14–15 weeks of age), *Siah2*^*-/-*^ females displayed a marked increase in diet-induced weight gain compared to wild type mice (Fig 6A). After 13 weeks on HFD (20–21 weeks of age), *Siah2*^*-/-*^ females were ~20% heavier compared to wild type females (Fig 6B), despite comparable food consumption (Fig 6C). *Siah2*^*-/-*^ males, on the other hand, ate and gained weight identically to wild type males (Fig 6A–6C). It is notable that the weight gain in *Siah2*^*-/-*^ females was comparable to males of both genotypes when the basal sex-difference in body weight was taken into account (Fig 6A and 6B).

The weight gain in SIAH2-deficient females was predominantly due to an increase in adiposity (Fig 6D). Notably, adipocytes of SIAH2-deficient mice of both sexes fed HFD were larger in size than those of wild type mice (S6A Fig), suggesting that the sex-specificity in obesity may be independent from SIAH2’s role in adipocyte development [40]. SIAH2 loss also altered how HFD impacted the regulation of serum cholesterol and triglyceride levels, selectively in females (S6B Fig). Finally, SIAH2 loss also led to the development of HFD-induced hepatic steatosis, a trait in which wild type females were protected (Fig 6E). Again, *Siah2*^*-/-*^ males did not show any differences from wild type males in these studies. We found that while HFD-induced obesity led to development of diabetic-like changes in glucose homeostasis in males of both genotypes, this was largely unaffected in *Siah2*^*-/-*^ females despite their increased obesity (S7 Fig). Taken together, these data confirm that SIAH2-deficient females, but not males, have deficits in the mechanisms regulating lipid and lipoprotein metabolism that normally appear to contribute to preventing systemic dislipidemia and diet-induced obesity.

## Discussion

Overall, we found a remarkable correspondence in the female-specificity of the effects of SIAH2 loss, from changes in gene expression to subsequent alterations in metabolism. Three complementary but different experimental paradigms examining gene expression in the liver all indicated that SIAH2 loss alters gene rhythmic expression selectively in females. Both RNAseq approaches suggested that regulation of rhythmic gene expression, particularly of genes involved in lipid and lipoprotein metabolism, was altered by SIAH2 loss only in females despite differences in experimental designs (single timepoint vs. around the clock). We found that SIAH2-deficiency does alter rhythmic gene expression in males, but the changes were distinct in every way from those in females. In females, the effects of SIAH2-deficiency were time-of-day specifc and focused predominantly on transcription factors and genes involved in regulating lipid and lipoprotein metabolism. The metabolic genes affected in females operate at multiple levels within lipid/lipoprotein metabolic pathways, suggesting SIAH2 loss impairs circadian output control of metabolism in females at many potential points. Importantly, the daily patterns of serum lipoproteins were likewise altered in a sex- and time-of-day dependent manner that coincided with the maximal changes in gene expression, providing both confirmation of the overall transcriptomics results and validating that the changes in gene expression are physiologically relevant.

The effects of SIAH2 loss on gene expression were nearly exclusive to two specific yet opposite times of day–mid-day and mid-night–where it flipped the expression of most nocturnally expessed genes to the day-time. This temporal precision suggests that SIAH2 is likely regulating a specific transcriptional pathway that regulates gene expression at these specific times-of-day. However, SIAH2 is a ubiquitin ligase with no known direct transcriptional activity. We therefore predict that it is regulating one or more transcription regulators in both a female- and a time-of-day specific manner. Also, although degradation is not the only mode of regulation possible by ubiquitin ligases, it provides an attractive potential explanation for our data. For example, SIAH2 loss could lead to increased/prolonged expression of genes due to elevated/prolonged function of a female specific ‘daytime activator’ (Fig 6F). This would imply that SIAH2 may normally function to limit the activity of this ‘daytime activator’. This ‘daytime activator’ may also drive rhythmic expression of one or more ‘nighttime repressors’ in a regulatory circuit or loop that go on to repress target genes at the night. In this way, the increased ‘daytime activator’ in the absence of SIAH2 results in increased expression of ‘nighttime repressors’, causing the large shift in timing of expression of genes from night-to-day. It is also possible that SIAH2 may target ‘nighttime repressors’ for degradation as well (Fig 3F). Degradation of these factors may not be the only mechanism by which SIAH2 is regulating their activity, but regardless of SIAH2’s specific action, it seems very likely that it is acting on substrates that are also female-specific. Furthermore, since SIAH2 selectvely regulates the rhythmic pattern of gene expression, we surmise that these unknown daytime and nighttime transcriptional factors likely comprises an inherent transcriptional output mechanism used by the circadian system to regulate rhythmic expression of ‘output’ genes. At this point, our data suggests these factors are distinct from known sexually dimorphic pathways in the liver, although their involvement cannot be completely excluded. Nonetheless, identifying these potential transcriptional regulators is of high importance as this overall mechanism is operating only in females, providing a strong indication that sex-differences are inherently integrated with the circadian clockwork to drive molecular and physiological rhythms for sex-specific purposes.

Perhaps the most obvious candidates for these SIAH2 substrates are transcriptional regulators that are differentially regulated by the circadian clock between males and females in wild type mice (e.g. transcription factors that are only rhythmic in females). Indeed, our data from wild type livers suggest there are more than 350 transcription factors or cofactors with different rhythmic expression profiles in males and females (see S3C Fig and S4 Dataset). Transcription factor binding site enrichment analysis [41] of the geneset altered by SIAH2 loss in females suggests members of the C2N2-type zinc-finger and ETS families may be the best candidates responsible for the changes in rhythmic gene expression. Combining these two analyses narrows this list of candiates to roughly 40 mouse transcription regulators that, with one exception, have not been linked to SIAH2 regulation or sexually dimorphic gene expression so far.

The gene encoding the androgen receptor (Ar) is the exception in this group. However, studies of AR function in circadian rhythmicity and AR-SIAH2 interactions predict there would be a stronger overall effect of SIAH2 loss in males instead of females. ARs can have a strong role in reguling lipid metabolism in the livers of both sexes, although its role in females may only occur under extra-physiological hormonal conditions [42]. The AR-SIAH2 interaction has been most extensively studied in prostate cancer where it regulates hormone sensitivity of tumor cells in conjuction with NCoR1[43], another SIAH2 substrate that also interacts with REVERBα [10,44]. AR activity also regulates SIAH2 E3 ligase activity in these cells [45], suggesting complex bi-directional regulatory interactions. AR activity in the SCN is also well known to modulate circadian behavior, but these effects are limited to male mice [46]. Similarly, in the liver, expression of the REVERBα and AR target gene *Elovl3* is expressed at ~100 fold higher levels in male livers compared to female livers [47] and its expression is not drastically affected by SIAH2 loss in either sex. Thus, although there are several intriguing interaction points between SIAH2, AR and NCoR1/REVERBα that might make AR an exciting candidate for the effects observed in males, we are looking for candidates that may be much more likely to produce the female-specific phenotypes we found. SIAH2 may interact with estrogen signaling in breast cancer cells in an analogous manner to AR in prostate cancer cells [48–50]. However, as noted above, our gene expression data does not suggest estrogen signaling is preferentially impacted by SIAH2 loss, dampening likelihood that this mechanism is involved. Moreover, the SIAH2-dependent changes in expression appear distinct from simply ‘masculinizing’ the female livers, although female *Siah2*^*-/-*^ mice had metabolisms that could be construed as masculine. At this point, it also does not seem likely that this role of SIAH2 is a result of changes in global REVERBα and/or NCOR1 stability, and does not appear to involve dimorphic growth hormone signaling pathways. Interestingly, juvenile (4 week old) *Siah2*^*-/-*^ mice fed HFD have also been shown to have sex-specific metabolic traits [51] that are somewhat different than those we observed, suggesting possible complex interactions between SIAH2, diet and puberty. Thus, although we cannot completely exclude these factors, or sex hormones in general, there is little evidence to suggest their potential misregulation is the main source of the female-specific phenotypes we have observed.

Regardless of how SIAH2 is acting, our data suggest that SIAH2 is a key component of an unexpected and unknown female-specific circadian clockwork mechanism that links circadian timekeeping to outputs that regulate rhythms in metabolism (Fig 6F). In the liver, this mechanism appears to be important for control of lipid metabolism, aligning daily metabolic rhythms in females to their behavior across the day. In this way, this novel SIAH2-dependent circadian mechanism may contribute to resilience against diet-induced obesity in females and the overall sexual dimorphism in metabolism. In addition, these findings imply that the circadian clockwork may drive gene expression and physiological rhythms using different molecular pathways in males and females. What these mechanisms are, however, still need to be discovered. Nonetheless, the sex-differences in these circadian mechanisms are essential to recognize and decipher as they may contribute to how males and females cope differently with circadian clock-related disorders [52–56] and possibly other fundamental physiological differences existing between males and females.

## Materials and methods

### Ethics statement

All animal studies were approved by the Institutional Animal Care and Use Committee of Morehouse School of Medicine in accordance with the United States Public Health Service Policy on Humane Care and Use of Laboratory Animals (protocol numbers 12–19, 18–14, 21–14).

### Animals and diets

Wild type and *Siah2*^*-/-*^mice maintained on a C57Bl6 background [18], kindly provided by Dr. Andreas Moller (QIMR Berghofer) and David Bowtell (Peter MacCallum Cancer Institute) were bred and maintained in our animal facility at Morehouse School of Medicine. After an initial cross to C57Bl6 mice, heterozygotes were intercrossed, and homozygous wild type and *Siah2*^*-/-*^ mice were used to establish related lines. All mice had *ad libitum* access to normal chow (PicoLab Laboratory Rodent Diet 5L0D, LabDiet, St. Louis MO, USA) and water cycle unless otherwise stated. With the exception of examining circadian control of locomotor behavior (S4 Fig) all mice were housed in a 12-hour light:12-hour dark (12:12 LD) for experimentation to eliminate potential intra-animal variability that could be caused by freerunning circadian rhythms, and to maintain similar experimental conditions across all experimental paradigms. Breeding mice were fed breeding chow (PicoLab Mouse Diet 20 5058, LabDiet, St. Louis MO, USA) and all other mice were fed normal rodent chow unless otherwise indicated. All mice were divided into groups according to sex and genotype, and were age-matched between groups prior to experiments. For the metabolic studies, mice were individually housed and provided Research Diets D12451 (45% kCal fat) as a high-fat det (HFD) or the sucrose matched control diet (CD) D12450H, starting at 7–8 weeks of age; body weights and food consumption were monitored weekly thereafter. After 13 weeks on these diets, mice were subjected to additional tests (see below). At the indicated times, mice were euthanized using CO_2_ followed by decapitation to collect blood and tissues.

### Body composition and serum analyses

Body composition was performed on mice that were fasted for 6 hours (starting at ZT0, lights off) prior to sacrifice. Body composition was performed post-mortem using MRI at the NIH University of Cincinnati Mouse Metabolic Phenotyping Center (MMPC). Serum was collected following euthanasia with CO_2_ and decapitation and samples were sent for analysis of insulin, total cholesterol, triglyceride, phospholipid and non-esterified fatty acid levels at the same facility. Sex hormones were measured from serum collected single-housed females in proestrus, as determined by cytological examination of vaginal lavages [57] and submitted to Ligand Assay & Analysis Core at the University of Virginia (https://med.virginia.edu/research-in-reproduction/ligand-assay-analysis-core) for measurements of LH/FSH (mouse/rat multiplex), estradiol (mouse/rat) and progesterone (mouse/rat) levels.

### Glucose and insulin tolerance tests

Mice were weighed at ZT0 and then fasted for 4 hours after which the fasting glucose levels were taken. Mice were then injected intraperitoneally with either 1.5 mg/gram body weight glucose solution (20% in 0.9% NaCl) or 0.75 IU of insulin per gram body weight (in 0.9% NaCl) and blood glucose levels were measured by drawing blood from the tail 20, 40, and 100 minutes post-injection using a Contour Blood glucose monitoring system (Bayer) as we have done previously [58]. Mice were first subjected to glucose tolerance tested, allowed to recover for 1 week before insulin tolerance testing.

### Liver and adipose staining

Oil Red O staining was performed on 16-micron frozen liver sections from wild type or *Siah2*^*-/-*^, male or female mice fed HFD using the Abcam Oil Red O Kit (ab150678), according to the manufacturer’s instructions. The stained sections were mounted and imaged at a magnification of 40x. Perigonadal white adipose tissue was dissected from the indicated mice, stored in a 15% sucrose at 4°C, and prepared for whole-mount staining [59]. Briefly, approximately 4mm x 4mm x 2mm sections of adipose tissue were washed in PBS for 10 minutes then stained sequentially with DAPI (Thermofisher; D1306) and Cell Mask Orange (ThermoFisher; C10045) for 1 hour each, with PBS washes between. Images and measurements were obtained using a spinning disc confocal microscopy system and Slidebook 6 (Intelligent Imaging Innovation, Denver CO, USA).

### RNA isolation and quantitative real-time PCR

Liver samples from *Siah2*^*-/-*^and wild type mice were collected every 3 hours over 24 hours (n = 3 per genotype and time point). Total RNA was extracted from livers using Trizol reagent (Invitrogen) per manufacturer’s instructions. Approximately one microgram aliquots of total RNA were reverse transcribed and subjected to quantitative PCR using Applied Biosystems cDNA kit (Thermo Fisher) and SsoAdvanced SYBR GREEN qPCR mix (Bio-Rad) respectively. Samples were run using the CFX96 Touch Real-Time PCR Detection System (running version 3.1 of the CFX Manager Software; Bio-Rad) and data all normalized to Gapdh and plotted relative to the time-independent average of the wild type samples using the 2^-ΔΔCt^ method. The sequences of the primers used were as follows:

m*GAPDH*: forward: AGACAGCCGCATCTTCTTGT, reverse: CTTGCCGTGGGTAGAGTCAT; mNr1d1: forward: CCCTGGACTCCAATAACAACACA, reverse: GCCATTGGAGCTGTCACTGTAG; *mArntl*: forward: AACCTTCCCGCAGCTAACAG, reverse: AGTCCTCTTTGGGCCACCTT; *mPer2*: forward: GAAAGCTGTCACCACCATAGA, reverse: AACTCGCACTTCCTTTTCAGG; *mDbp*: forward: GGAACTGAAGCCTCAACCAAT, reverse: CTCCGGCTCCAGTACTTCTCA; *mCry1*: forward: TGAGGCAAGCAGACTGAATATTG, reverse: CCTCTGTACCGGGAAAGCTG. QuantiTect primer assays for *Mir122* precursor, *Fasn* and *Acaca* were purchased from Qiagen

### Transcript profiling

We performed two different transcript profiling experiments using slightly different approaches. For the data shown in Fig 1, livers were collected at ZT10 from mice of both sexes and genotypes at ~20 weeks of age, and total RNA was extracted using Trizol, followed by clean up using RNeasy kits (Qiagen). Transcripts were profiled using the Lexogen QuantSeq 3’ mRNA kit to ‘count’ transcript abundance. Library prep and sequencing was performed by Omega Bioservices (Norcross, GA, USA) using the Illumina HiSeqX10 platform. Data were trimmed, mapped and analyzed using Illumina’s BaseSpace platform. Differential expression was determined using RNAexpress and the DESeq2 method. Transcripts flagged as having an ‘outlier’ or ‘low expression’ were excluded from consideration, resulting in 8405 and 8709 total transcripts for comparison in male or females respectively. Full count data and DESeq2 results are provided in S1 Dataset. Enrichment of rhythmic genes in these data was determined using the "Mouse 1.0ST Liver" and "Mouse Liver 48 hour Hughes 2009" datasets (http://circadb.hogeneschlab.org/mouse), with a probabilty cut-off value at q<0.05 using the gene symbols for all 513 differentially expressed genes as search terms.

For the diurnal transcriptomics, livers were isolated from *Siah2*^*-/-*^ and wild type mice maintained on normal chow at 3-hour intervals across a 12:12 LD cycle. Total RNA was isolated and cleaned-up as described above, and equal amounts were pooled from livers from 3 mice/time/genotype/sex for RNAseq. RNAs were converted into sequencing libraries by using Illumina TruSeq stranded mRNA Library Prep kits and sequenced by Omega BioServices (Norcross, GA) using the Illumina HiSeqX10 platform. Samples were sequenced to a depth >35 million 150bp X 150bp paired end reads. The reads were mapped to the mouse MGSCv39-mm9 genome using Tophat 2.1.0. Expression levels were assessed using Cufflinks 2.2.1, which calculates raw counts and the number of fragments per kilobase per million (FPKM). Low expression genes were filtered out if the sum of their raw count was less than 100 across the time points in all 4 groups (male wild type, male *Siah2*^*-/-*^, female wild type and female *Siah2*^*-/-*^). Count data was used for DESeq2 (https://yanli.shinyapps.io/DEApp/) (S3A Fig), and FPKM was used for all other analyses. Raw (FASTQ) and processed data (counts, FPKM) are available at NCBI Geo, accession number GSE182836.

### Time series analysis for circadian cycling

MetaCycle::meta2d (ver.1.2.0; https://CRAN.R-project.org/package=MetaCycle) was used to detect circadian transcripts with the default settings and period length set to 20 for ‘minper’ and 28 for ‘maxper’ [60]. Data from one cycle was concatenated to create a 48 hour time series for analyses using JTK cycle [61] to identify cycling transcripts and their peak expression phases and DODR ver.0.99.2 [62] (https://CRAN.R-project.org/package=DODR) to directly compare gene expression profiles between sexes/genotypes. We acknowledge that concatenating the data like this likely increases the false-positive rates in both algorithms. However it also greatly reduces the false-negative rate that especially JTK can have when analysing a single cycle; JTK was developed to operate more effectively with two cycles of data. False negatives for rhythmicity are much more problematic for our analyses as these would likely falsely amplify the differences between groups–something we sought to minimize as much as possible. To help limit the overall effect of false-positives, we combined results from 2–3 measures (i.e. rhythmicity, phase, meta.p) from both analyses to define differences (described below). It should be noted that performing these analyses on non-concatenated data produces the same overall proportionality in the results–SIAH2-deficient females still have ~60% more rhythmically expressed genes than wild type at all cut-off levels (S2A Fig), with similar changes in phase distribution (S2B and S2C Fig), but with fewer numbers of genes in each category. The peak times (i.e. phase) were derived using JTK cycle and imported into Oriana (version 3, Kovach Computing, Anglesey, Wales UK) to produce Raleigh plots and GraphPad Prism (v7 or later; GraphPad Software, SanDiego CA, USA) for further analyses. Heat maps were generated using an R code (https://github.com/gangwug/SRBR_SMTSAworkshop/blob/master/R/fig.R).

The goal of JTK-cycle is to identify rhythmic transcripts within a dataset but does not perform direct comparisons between datasets. DODR identifies overall differences between time-series datasets, including phase, amplitude and overall abundance, but does not assess circadian-like rhythmicity per se, thus can identify changes even in transcripts that are not rhythmic in either dataset being compared. Therefore, we used JTK cycle parameters to define rhythmicity and peak phase, and DODR to substantiate differences/lack of differences between groups, as depicted in the Table 1 below (bold font indicates key difference).

**Table 1:** Statistical criteria used to filter genes into the categories listed in the left column. Bold are the key factor(s) for each category.

	Group 1 JTK adjP value	Group 2 JTK adjP value	DODR meta.p	Phase difference
**Rhythmic in both, unchanged**	< 0.05	< 0.1	< 0.01	**< 6 hours**
**Rhythmic in both, phase change**	< 0.05	< 0.1	Not used	**> 6 hours**
**Rhythmic in both, other difference**	< 0.05	< 0.1	**> 0.01**	< 6 hours
**Gained Rhythm in KO/ Females only**	< 0.05	**> 0.1**	**> 0.01**	< 6 hours
**Lost Rhythm in KO/ Males only**	**> 0.1**	< 0.05	**> 0.01**	< 6 hours

We chose these JTK adjP value cutoffs in attempt to prevent overestimating differences between groups, while still including genes with less robust rhythms. Using the same cutoffs for both groups increases the number of different genes between groups, but reducing the cutoffs proportionally reduces the numbers of genes in each category, but the differences between groups remain proportional (i.e. there are still more differences in females than males). DODR was not used to support phase changes, but meta.p > 0.01 for 85% of genes with SIAH2-loss induced phases differences in females (Fig 4A), 75% of those changes in males (Fig 4A) and 82% of sexually dimorphic phase changes (S3C Fig) genes (**see S2–S4 Datasets**). In addition, we found small subsets of genes we classified as rhythmic across both groups, but DODR identified a difference other than a 6-hour phase change but were not closely examined but are denoted in **S2–S4 Datasets**. Of note, the differences in gene expression observed via qRT-PCR (Fig 1) were not readily detectable in the RNAseq data due to sample pooling and the differences in analysis methodologies. All of the differentially expressed genes were combined and subjected to Gene Ontology analyses (http://geneontology.org). Top relevant child terms for biological processes were selected after sorting by decreasing order of fold enrichment and increasing order of p-values.

### Locomotor and feeding behavior

Wheel running locomotor activity was recorded and analyzed as described previously [63]. For feeding behavior, mice were fed ad-libitum with normal chow and eating behavior was recorded for 8 days using infrared video cameras (ZOSI 720p CVI TVI, ZosiTech, Zhuhai city, China) connected to a TigerSecu 8 Channel DVR Security Video Recording System. Videos were analyzed using Noldus EthoVision XT (v14, Noldus, Leesburg VA, USA), and feeding behavior was coded in 1 min bins using the following criteria: 1) the mouse took food from the feeder with its mouth or, 2) the mouse moved food in the feeder with its mouth; either behavior having persisted for 3 seconds or more. When eating behavior occurred, that 1 min bin was coded as “1”. One-minute bins without any feeding behavior were coded as “0”. One-minute bins were summed for each hour and averaged across the 8-day recording according to ZT hour in 1-hour intervals for each mouse, and then normalized to the within animal mean of its total daily feeding activity.

### Statistical analyses

Except where noted above, all graphs and statistical analyses were generated using Graphpad Prism (v7 or later; GraphPad Software, SanDiego CA, USA). Statistical analyses performed were typically two-way ANOVAs, examining *sex* x *genotype* or *genotype* x *diet* interactions, followed by Sidak’s multiple comparison test, unless otherwise indicated. Specific p-values for the two-way interaction test are indicted in figures or legends if significant. Relevant F values and degrees of freedom are also reported as ‘F (DFn, DFd) = [value]’ or t = [value], df = [value]. Differences were considered significant if p<0.05, unless otherwise indicated. Sample sizes were determined *a priori* based on conventions in the literature, taking into consideration technical limitations (i.e. 96 samples in a 96 well qPCR machine), effect size/type and number of animals to be utilized and are indicated with each figure legend as appropriate.

## Supporting information

S1 Dataset The QuantSeq results for all transcripts detected. Official gene symbol, counts for each individual, means for each genotype and the results of DESeq2 analysis. Data are sorted by *padj* value. The differential genes in ‘Circadian Rhythm’ DAVID term are highlighted in red font. Rows highlighted in grey are those (based on gene ID) that also display altered rhythmcity in SIAH2-deficient mice (from S2 and S3 Datasets).
(XLSX)

S2 Dataset The genes/NM identifiers, FPKM data, and the JTK-cycle and DODR results from comparing wildtype and *Siah2*^*-/-*^ females. (XLSX)

S3 Dataset The genes/NM identifiers, FPKM data, and the JTK-cycle and DODR results from comparing wildtype and *Siah2*^*-/-*^ males. (XLSX)

S4 Dataset The genes/NM identifiers, FPKM data, and the JTK-cycle and DODR results from comparing wild type males and wild type females. (XLSX)

S5 Dataset The genes with time-independent changes in gene expression, due to either SIAH2 loss (in males and females) or sex. DESeq2 results are included.
(XLSX)

S1 Fig SIAH2 loss does not drastically alter REV-ERBα stability in livers. Representative (of 2 independent experiments) western blots of pooled liver samples (n = 3 mice/pool) collected around the clock (time 12 = lights out). The bar graphs are the densitometric quantification of the blots shown.
(EPS)

S2 Fig Analysis of single-day data profiles. **A**. Plot of the number of rhythmic genes based on different JTK cutoffs in each group. Wild type males and females, as well as SIAH2 KO males all have similar numbers of rhythmic genes across cutoffs; SIAH2 KO females have ~50–60% more rhythmically expressed genes at any cutoff. **B**. Heatmaps of the genes rhythmic at AdjP <0.05 for each group, plotted independently. **C**. Frequency distribution of peak expression timing for the genes plotted in B. Numbers around the clock face are hours, ZT time (light and dark are indicated by shading). Red lines and error bars are the mean peak timing +/- 95% CI for the entire population, r = radii of the circular plots in number of genes.
(EPS)

S3 Fig SIAH2 loss does not drastically alter time-independent gene expression or preferentially target common sexually dimorphic pathways. **(A)** RNAseq data were combined across timepoints according to sex/genotype, treating timepoints as biological replicates (n = 8 per sex/genotype) and the effects on SIAH2 loss on gene expression within each sex (left), or between sexes in wild type mice (right) were compared using DESeq2. Both volcano plots only show the genes that were significantly different (pAdj <0.05), and are plotted on the same y-axis for comparison. The sex symbols on the right plot indicate higher expression in female or male livers, respectively. Genes for both are listed in **S5 Dataset. (B)** SIAH2 expression itself is not robustly sexually dimorphic. RNAseq FPKM of SIAH2 in wild type male and female livers. Overall there was no detectable difference in expression (DoDR meta p >0.05, DESeq2 padj>0.05) despite evidence that Siah2 may be rhythmically expressed in females, but not males (JTK p values are listed). **(C)** Comparison of the effects on diurnal gene expression of SIAH2 loss in males and females and the effect of sex in wild type mice, categorized as described in the Materials and Methods. Some of these data are replotted from Fig 2 for comparison. Venn diagrams on the right depict the overlap in genes with dimorphic rhythmic expression with those differentially expressed by SIAH2 loss in male or female livers, across the categories shown on the left. See also **S2–S4 Datasets. (D)** We collected serum from females in proestrus sacrificed between ZT 7–14 for measurement of the indicated hormones (LH levels were below level of detection). Mean +/- SEM (n = 7 each, except n = 5 for wild type progesterone) hormone levels are shown. p > 0.5 for each, unpaired two-tailed t-tests. (**E)** Litter sizes for last 50/49 litters obtained from 18/19 homozygous wildtype/homozygous *Siah2*^*-/-*^ females (respectively), bred with males of like genotypes. Individual data points and mean +/- SEM are shown (p = 0.3, t = 1.038, df = 97, two-tailed t-test). **(F).** *Left-* Venn diagram depicting the overlap between genes regulated by SIAH2 loss and estrogen signaling in female livers (Ref. 26). *Right—*Proportions of transcripts whose expression was altered by SIAH2 loss in female livers. SIAH2 loss altered the expression of 3,540/22,002 total genes, or ca. 16% of the total genes examined. SIAH2 loss altered a similar proportion (ca. 18%) of estrogen responsive genes suggesting that SIAH2 loss is not selectively impacting estrogen-signaling. Thus, the effects of SIAH2 loss on rhythms is unlikely to occur via mis-regulation of estrogen signaling. **(G)** RNAseq profiles for representative genes encoding factors involved in mediating sexual-dimorphic gene expression in the liver.
(EPS)

S4 Fig Behavioral profiles are not altered by SIAH2 loss in females. **A**) Representative double-plotted actograms of wheel running behavior in wild type or *Siah2*^*-/-*^ mice. The first 8 days of the recordings (indicated by the alternating white/gray shading) were done on a 12:12LD cycle, followed by constant darkness (solid gray shading). Individual and mean +/- SEM data obtained from mice of both genotypes and sexes are shown on the right, combined from animals of both sexes and genotypes run in two independent experiments (n = 9 wild type males, 9 wild type females, 14 *Siah2*^*-/-*^ males, and 8 *Siah2*^*-/-*^ females). SIAH2 loss did not significantly alter the behavioral circadian periods within either sex (p>0.9, two-tailed t-test, effect sizes of either genotype was less than 0.006 hours). **(B)** Diurnal feeding behavior of wild type (n = 5) and *Siah2*^*-/-*^ (n = 4) female mice on normal chow. Each point is the mean of the average 8-day profile produced by each animal, and the error bars (SEM) indicate the inter-animal variability at each time point. Two-way ANOVA did not detect a significant interaction between genotype and time that would support a change in the daily feeding pattern (p = 0.4751 for interaction, F (23, 161) = 0.9947).
(EPS)

S5 Fig Effects of SIAH2 loss on serum lipoproteins are female specific. Serum was harvested from mice sacrificed between ZT3-6 and assayed for total cholesterol (**A**) and phospholipids (**B**). Data are the mean +/- SEM, n = 15 combined from three independent trials. Data are normalized to wild type to eliminate trial-to-trial differences in overall levels. * = significantly different from wild type, p <0.05, two-sided t-test.
(EPS)

S6 Fig Effects of HFD on adipocyte size and serum lipid regulation in SIAH2-deficient mice. (**A**) Left–Representative images of adipose samples obtained from mice fed HFD for >13 weeks and stained with Cell Mask Orange (red) and DAPI (blue) to visualize cell membranes and nuclei, respectively. Scale bar = 50 microns. Right–quantitation of 100 cell diameters measured per mouse (mean +/- sem, n = 3 mice/group). * p = 0.018 (F (1, 8) = 8.836), main effect of genotype, two-way ANOVA. **(B)** Serum triglycerides (upper) or total cholesterol (lower) in response to 13 weeks of feeding CD (dark bars) or HFD (light bars). Sample were collected at ZT10 after a 6 hour fast. Mean +/- SEM are shown (n = 5 mice sampled per group, except n = 4 for SIAH2 KO males or females fed CD). * p = 0.048 (t = 2.05, df = 15), ** p = 0.009 (t = 30342, df = 15), *** p = 0.043 (t = 2.563, df = 15), Sidak’s multiple comparisons test.
(EPS)

S7 Fig Obese SIAH2-deficient females do not show strong diabetic-like phenotypes. Fasting serum glucose and insulin levels, as well as glucose and insulin tolerance (GTT and ITT, respectively) in males **(A)** and females **(B)**. All animals were fasted for 4 hours (starting at ZT0) before testing. Data are all means +/- sem, n = 5–7 mice per group, except n = 4 for wild type CD males and wild type HFD females. * = p<0.03, ** p = 0.0009 for time x diet interaction (F (3, 72) = 6.140), three-way ANOVA), and main effect of diet (p<0.0001, F (1, 72) = 121.0) but no three-way interaction (p = 0.79, F (3, 72) = 0.3480), *** main effects of diet (p<0.0001, F (1, 72) = 68.48") and genotype (p = 0.0043, F (1, 72) = 8.688) only, no three-way interaction (p = 0.6376, F (3, 72) = 0.5684). ^#^ p = 0.039, t = 2.605, df = 15 and ^##^ p = 0.037, t = 2.64, df = 15 using Sidak’s multiple comparison test.
(EPS)
